# Examining the impact of changes in school tobacco control policies and programs on current smoking and susceptibility to future smoking among youth in the first two years of the COMPASS study: looking back to move forward

**DOI:** 10.1186/s12971-015-0031-1

**Published:** 2015-03-30

**Authors:** Scott T Leatherdale, Adam Cole

**Affiliations:** School of Public Health and Health Systems, University of Waterloo, 200 University Avenue, Waterloo, ON N2L 3G1 Canada

**Keywords:** Tobacco, Smoking, Policy, Program, Intervention evaluation, Youth, Adolescence, Schools

## Abstract

**Background:**

School-based prevention activities continue to be an important tobacco control resource, however there is little guidance for school-based tobacco control programming within Ontario. The objective of this study is to identify real-world changes in school-based tobacco control programs or policies in the COMPASS study and examine of those interventions (natural experiments) had any impact on the school-level prevalence of smoking susceptibility and current smoking over time.

**Methods:**

This paper uses longitudinal school-level smoking behaviour data from Year 1 (Y_1_: 2012–13) and Year 2 (Y_2_: 2013–14) of the COMPASS study. Changes to school-level tobacco control programs and policies were measured using the COMPASS School Programs and Policies Questionnaire and knowledge broker follow-up interviews. Quasi-experimental tests of proportion and difference-in-difference models were used to evaluate the impact of the interventions identified between Y_1_ and Y_2_ on school-level prevalence of smoking susceptibility among never smokers and current smoking.

**Results:**

Between Y_1_ and Y_2_, 17 schools reported a change in their tobacco control programming or policies. In four of the intervention schools, the increase in the within-school prevalence of susceptible never smokers between Y_1_ and Y_2_ was significantly greater than the natural change observed in the control schools. In five of the intervention schools, the decrease in the within-school prevalence of current smokers between Y_1_ and Y_2_ was significantly greater than the natural change observed in the control schools. Only two of the new interventions evaluated (both focused on policies of progressive punishment for students caught smoking on school property), were associated with significant desirable changes in both smoking susceptibility and current smoking between Y_1_ and Y_2_.

**Discussion:**

Interventions specific to effective and enforced tobacco control were the most common and consistently had the desired impact on the school-level prevalence of smoking susceptibility and current smoking. Due to the variation in the types of interventions implemented and their effectiveness, additional evaluation evidence is necessary to determine the most successful activities and contexts among individual students. The results presented here highlight which of these real-world promising interventions should be further evaluated using the longitudinal individual-level data in COMPASS over time.

## Background

There is substantial evidence highlighting how youth progress through a variety of stages during the process of becoming a smoker that include transitioning from being a non-smokers who is contemplating trying a cigarette (also known as being susceptible to smoking) to smoking on a regular basis [1]. In the Canadian context, recent national evidence suggests that among Canadian youth in grades 9 to 12, 29.3% were susceptible never smokers (i.e., the youth most likely to become future smokers but who are not yet smoking) [2], and 8.9% were current smokers [3]. Despite substantial declines in youth smoking in Canada in recent decades, smoking continues to remain as one of the leading public health issues pertaining to the future health of Canadian youth.

The Theory of Triadic Influence (TTI) [4] suggests that youth smoking behaviour is influenced by a variety of factors from multiple levels of influence, ranging from proximal causes (e.g., individual student psychosocial and behavioural characteristics), distal causes (e.g., the program and policy environment in a school), to ultimate causes (e.g., cultural and biological determinants). As such, to improve the understanding of youth smoking behaviour and how to best intervene, we require evidence pertaining all of these levels of influence and how they interact [4]. There is a substantial body of evidence pertaining to both the proximal individual-level factors and overarching cultural and biological determinants associated with youth tobacco use [5]. However, despite evidence to suggest that the school environment is independently associated with both susceptibility to smoking among never smokers [2,6] and current smoking [7,8], our understanding of how to effectively intervene within the school context is limited [9,10]. For instance, a recent review of school-based tobacco control policies concluded that the evidence of the effectiveness of school anti-tobacco policies is weak and inconclusive [9]. As such, while school-based prevention activities continue to be an important tobacco control resource [5], it is also a domain where the research evidence to inform future practice is clearly lacking.

In 2006, the Government of Ontario implemented the Smoke-Free Ontario Strategy (SFO), as an innovative multi-level comprehensive tobacco control strategy in the province designed to eliminate tobacco-related morbidity and mortality [11]. According to the Government of Ontario, funding for the SFO has ranged between $40-$60 million annually to supplement and enhance comprehensive tobacco control programming, with one of the three priority areas being to prevent experimentation and escalation of tobacco use among youth [11]. Current SFO strategies are classified under eight headings: media and social marketing interventions, interventions to address smoking in the movies and video games, effective and enforced tobacco control policies, industry marketing and promotion interventions, cessation interventions, tobacco denormalization interventions, aligned and coordinated interventions, and targeted prevention interventions [12]. Considering the majority of Ontario youth attend secondary school, the school environment represents an equitable context for altering youth smoking attitudes and behaviours. However, there currently appears to be only one school-based SFO pilot project taking place within a small sample of select Ontario secondary schools (the intervention started in 2013), and the majority of the current SFO strategies designed to prevent youth smoking occur outside of the school context or control of schools (e.g., interventions to promote smoke-free movies, point-of-sale restrictions, increasing tobacco taxes, etc.) [13]. The lack of guidance for school-based tobacco control programming within Ontario leaves schools with the task of either identifying and implementing existing evidence-based programs (which may not be appropriate or feasible in their school context), or they are forced to develop their own unique programs or policies which are often not evidence-based [14]. Moving forward, it would be beneficial to determine if schools are implementing any new tobacco control interventions, to identify what those tobacco interventions are, and to start to generate some practice-based evidence of their real-world effectiveness by evaluating such natural experiments [15,16]. Considering the lack of evidence pertaining to what school-based tobacco control programs are effective under which conditions [9,10], evidence from such natural experiments can provide a timely and robust addition to our current limited evidence-base.

Data collection and feedback systems are beneficial for improving the capacity to integrate research, evaluation, policy, and practice within school-based tobacco control programming [11,14,17]. In Ontario, the COMPASS study was designed to address that need [18]. As described in more detail elsewhere [18], the rigorous longitudinal quasi-experimental design of COMPASS allows researchers to evaluate how ongoing school-specific changes in school-based tobacco control programs or policies are related to changes smoking uptake and progression over time. COMPASS also facilitates action in tobacco control by annually providing each participating school with customized knowledge exchange tools and access to a knowledge broker to help connect them to relevant tobacco control prevention resources. COMPASS aligns well with the recent recommendation that tobacco control research take more of a systems science approach [19], where the research should better reflect the real-world, where policy changes are examined in terms of both desirable and potentially undesirable outcomes, and where external validity is valued. As such, the objective of this study is to examine if real-world changes in multiple school-based tobacco control programs or policies between Year 1 and Year 2 of the COMPASS study had any impact on either (a) increasing the school-level prevalence of never smokers who remain susceptible to smoking, or (b) decreasing the school-level prevalence of current smoking. In remaining true to a systems ethos, we are simultaneously evaluating the impact of multiple different school-based tobacco prevention programs within the paradigm of a natural experiment.

## Methods

### Design

COMPASS is a cohort study collecting hierarchical longitudinal data from a sample of grade 9 to 12 secondary school students and the schools they attend in Ontario and Alberta, Canada [18]. The current paper reports longitudinal school-level prevalence data using the student-level data from Year 1 (Y_1_: 2012–2013) and Year 2 (Y_2_: 2013–2014). A full description of the study methods is available in print [18] or online (www.compass.uwaterloo.ca).

### Participants

In Y_1_, 43 Ontario schools were purposefully recruited because they provided permission to use active information-passive consent parental permission protocols. In the active information-passive consent protocol, the parent(s) or guardian(s) of eligible students were mailed an information letter about the COMPASS study and were asked to contact the COMPASS recruitment coordinator using either the toll-free phone number or email address provided in the information letter, should they not want their child to participate. All eligible students whose parent(s) or guardian(s) did not contact the COMPASS team to withdraw their child were deemed eligible to participate. Students could decline to participate at any time. Students were not made aware of the specific data collection date ahead of time (i.e., to prevent at-risk youth from purposefully skipping school that day). A total of 30,147 students were enrolled in grades 9 to 12 in the 43 COMPASS secondary schools in Y_1_. Overall, 80.2% (n = 24,173) of eligible Y_1_ students completed the COMPASS student questionnaire in class time on the day of their schools scheduled data collection. Missing respondents resulted from field trips/absenteeism/classroom spares on the day and time of the survey (18.9%) and parental/student refusal (0.9%). An additional 252 students were deleted due to missing data for gender and grade resulting in a final sample of 23,921 Y_1_ respondents for this manuscript. In Y_2_, the sample included students from the same 43 Ontario schools. A total of 29,951 students were enrolled in grades 9 to 12 in the 43 COMPASS secondary schools in Y_2_. Overall, 78.2% (n = 23,424) of eligible Y_2_ students completed the COMPASS student questionnaire in class time on the day of their schools scheduled data collection. Missing respondents resulted from field trips/absenteeism/classroom spares on the day and time of the survey (20.9%) and parental/student refusal (0.9%). An additional 307 students were deleted due to missing data for gender and grade resulting in a final sample of 23,117 Y_2_ respondents for this manuscript. The Y_1_ and Y_2_ student data are not linked in this paper given the analytical approach we have used for this quasi-experimental evaluation of multiple simultaneously occurring real-world interventions. The promising interventions identified here can be further examined individually in more detail in future studies by linking the longitudinal individual-level data and evaluating their impact when controlling for important school-level and student-level characteristics.

### Data collection tools

The student-level questionnaire for COMPASS (C_q_) collects individual student data pertaining to multiple behavioural domains (e.g., tobacco use, obesity, physical activity, diet, substance use, etc.), correlates of the behaviours, and demographic characteristics. In each school, the C_q_ was used to collect within-school samples during class time. The C_q_ items have demonstrated reliability and validity for current smoking [20] and smoking susceptibility among never smokers [21].

Changes to school-level programs and policies related to tobacco control in the 43 schools between Y_1_ and Y_2_ were measured using the COMPASS School Programs and Policies Questionnaire (SPP). The SPP is a paper-based survey completed annually by the school administrator(s) most knowledgeable about the school program and policy environment within a school. The SPP was based on the previously validated Healthy School Planner tool [22], but modified to be shorter in length and to cover additional content domains. Specific to this manuscript, the SPP measures the presence or absence of relevant tobacco control programs and/or policies, changes to school policies, practices, or resources that related to tobacco control. The SPP also asks school administrators to rank various behavioural domains in terms of their priority for future prevention action within the school. The completed SPP from each school was collected by COMPASS staff at the time of their school’s student-level data collection along with copies of the relevant policy handbook(s) or rules for additional document review if required. COMPASS staff also follow up with each school to verify the information provided.

### Measures

#### Student-level measures

The operational definitions for the student-level measures used in this manuscript are consistent with previous research using validated measures [20,21]. Smoking susceptibility among never smokers (respondents who reported that they have never smoked a cigarette, not even a puff), was derived by three previously validated measures [21] which asked respondents: “Do you think in the future you might try smoking cigarettes?”; “If one of your best friends were to offer you a cigarette, would you smoke it?”; and, “At any time during the next year do you think you will smoke a cigarette?” Students responded to these questions on a 4-point Likert scale and students who answered definitely not to all three questions were considered non-susceptible; students were considered susceptible if they responded positively to at least one item [21]. Current smoking was measured by asking respondents, “Have you ever smoked 100 or more whole cigarettes in your life?” and “On how many of the last 30 days did you smoke one or more cigarettes?”. Consistent with previously validated measures of current smoking among youth [20], students who reported ever smoking 100 cigarettes and any smoking in the previous 30 days were classified as current smokers.

#### School-level measures

Using the student-level data from the C_q_, the prevalence of susceptible never smokers and current smokers was determined for each school at Y_1_ and Y_2_. Although it may appear counter-intuitive, for the analyses proposed within this manuscript, an increase in the school-level prevalence of never smokers who remain susceptible to smoking was seen as the desirable outcome as it suggests that among the never smoking youth who are at risk for smoking, fewer of them have actually transitioned to experimenting with smoking; despite remaining at-risk for future smoking, these students were still never smokers. For current smoking, a decrease in the school-level prevalence of current smokers over time is the desirable outcome.

Data from the SPP administered in Y_2_ were used to identify any changes to school tobacco control programs and policies between Y_1_ and Y_2_. The Y_2_ SPP asked administrators to report if there have been any changes to their school tobacco control practices and policies since last school year. They were specifically asked to consider and comment on: a) whether Y_1_ policies, practices, environment and relationships are still in place; and, b) whether any new policies, practices, environment changes or relationships were implemented. To further corroborate that any identified changes actually took place (or to verify that nothing changed), the COMPASS knowledge broker (a staff member who had ongoing contact with each participating school administrator) verified all of the tobacco control program and policy changes identified in the Y_2_ SPP. Data from the SPP were also used to determine the priority of tobacco control within the schools prevention programming priorities each year.

### Analyses

Consistent with recommendations for examining quasi-experimental research designs [23], tests of proportion (t-tests) were used to examine (a) the significance of changes in the school-level prevalence of susceptibility among never smokers and current smoking between Y_1_ and Y_2_ for each school that reported a change in tobacco control programming (*intervention schools*), and (b) the difference-in-difference changes for each intervention school relative to the sample of schools that reported no changes in school-level tobacco control programming (*control schools*). The different types of interventions identified were grouped into the appropriate SFO area classification: media and social marketing interventions, effective and enforced tobacco control policies, industry marketing and promotion interventions, cessation interventions, tobacco denormalization interventions, aligned and coordinated interventions, and targeted prevention interventions [12]. Given the quasi-experimental design and that the analyses are longitudinal at the school-level between Y_1_ and Y_2_ for this difference-in-difference modelling approach, these models did not need to control for other associated school-level characteristics (e.g., school-level socio-economic status, school location, etc.), as these school-level factors would not have varied within a school between Y_1_ and Y_2_. Consistent with previous recommendations [23], future research examining these promising interventions individually using the longitudinal student-level data would need to control for relevant school-level and student-level correlates. The statistical package SAS 9.4 was used for all analyses.

## Results

In Y_1_, 71.3% (n = 17,054) of the sample were classified as never smokers of which 27.2% (n = 4,645) were classified as susceptible to future smoking. In Y_2_, 71.6% (n = 16,550) of the sample were classified as never smokers of which 29.0% (n = 4,798) were classified as susceptible to future smoking. In Y_1_, 5.8% (n = 1,380) of the sample were classified as current smokers and in Y_2_, 6.2% (n = 1,425) of the sample were classified as current smokers. Between Y_1_ and Y_2_, a total of 17 schools reported a change in their tobacco control programming or policies whereas the remaining 26 schools reported that there were no changes in school-level tobacco control programming (*control schools*). In the 26 control schools, the mean prevalence of susceptible never smokers was 27.8% (range 18.7% to 39.9%) in Y_1_ and 29.8% (range 22.4% to 41.9%) in Y_2_, and the mean prevalence of current smoking was 7.0% (range 2.2% to 23.9%) in Y_1_ and 7.4% (range 1.9% to 19.7%) in Y_2_. The 2.0% change in the mean prevalence of susceptible never smokers in the control schools between Y_1_ and Y_2_ represents a 7.1% increase in smoking susceptibility among never smokers, while the 0.4% change in the mean prevalence of current smoking in the control schools between Y_1_ and Y_2_ represents a 5.6% increase in current smoking. In both Y_1_ and Y_2_, tobacco prevention programming was ranked as the third lowest prevention priority out of 10 possible priorities (none of the schools ranked tobacco as the top priority in Y_1_ whereas one school ranked tobacco as the top priority in Y_2_).

### Changes in tobacco control programs and policies

Between Y_1_ and Y_2_, 17 changes to school-based tobacco control programs and policies (*interventions*) were identified.

**Figure 1:**
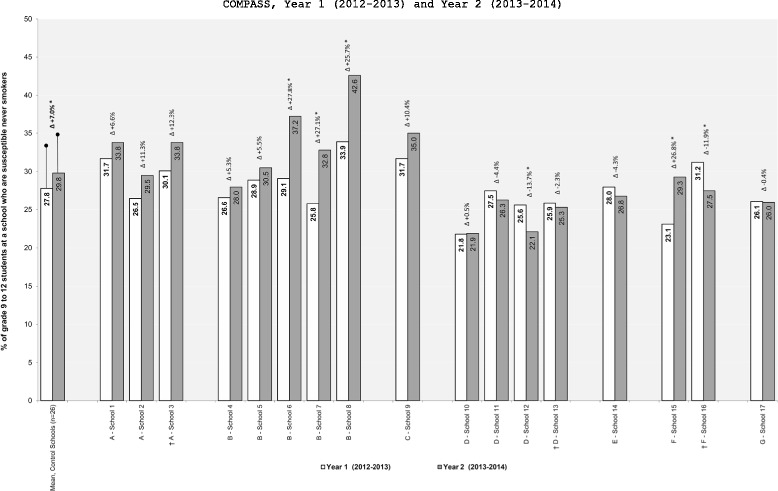
**Changes in school-level prevalence of susceptible never smokers as a function of changes in school-level tobacco control programs or policies.** † Indicates a program or policy was stopped or removed. * p<0.05. A - Media and Social Marketing Interventions; B - Effective and Enforced Tobacco Control Policies; C -Industry Marketing and Promotion Intervention; D - Cessation Interventions; E - Tobacco Denormalization Intervention; F - Aligned and Coordinated Interventions (Staff Training); G - Targeted Prevention Intervention;Control Schools reported no changes to their tobacco control programs and policies between Y_1_ and Y_2_.

**Figure 2:**
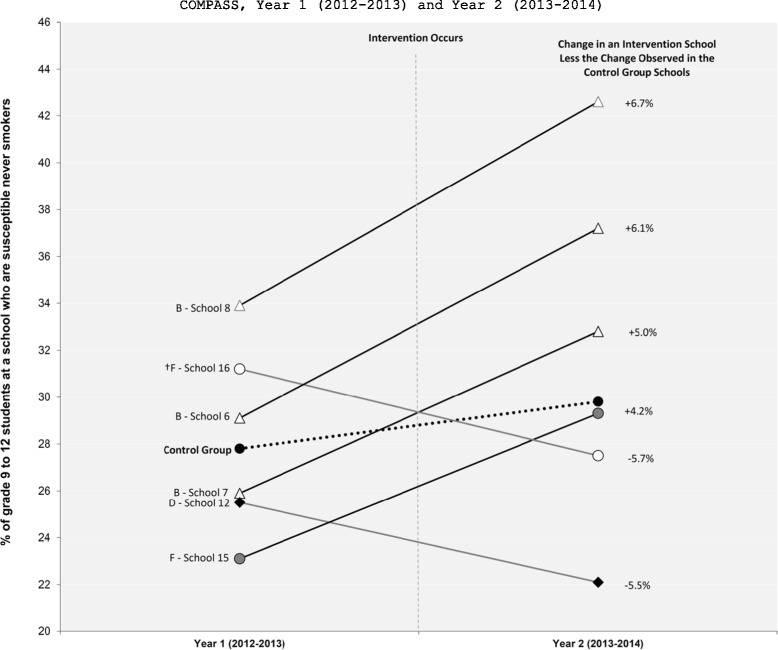
**Difference-in-difference results of the school-level tobacco control programs or policies with significant pre-post differences in the school-level prevalence of susceptible never smokers.** † Indicates a program or policy was stopped or removed. B - Effective and Enforced Tobacco Control Policies; D - Cessation Interventions; F - Aligned and Coordinated Interventions (Staff Training). Control Schools reported no changes to their tobacco control programs and policies between Y_1_ and Y_2_.

**Figure 3:**
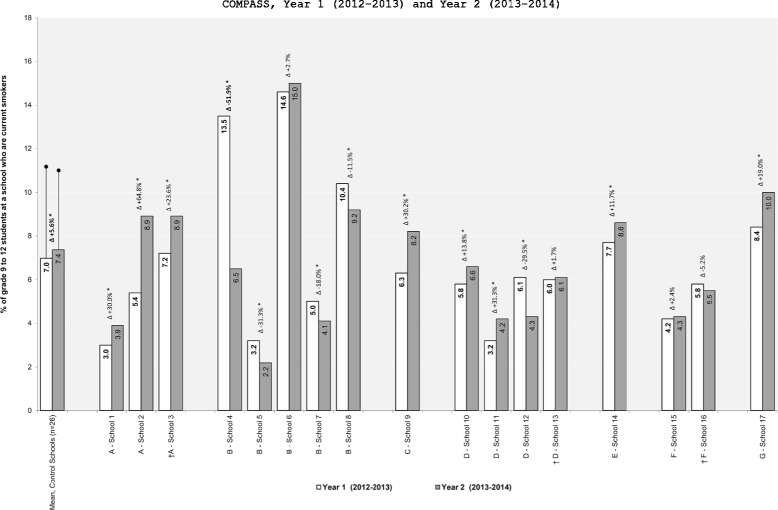
**Changes in school-level prevalence of current smokers as a function of changes in school-level tobacco control programs or policies.** † Indicates a program or policy was stopped or removed. * p<0.05. A - Media and Social Marketing Interventions; B - Effective and Enforced Tobacco Control Policies; C - Industry Marketing and Promotion Intervention; D - Cessation Interventions; E - Tobacco Denormalization Intervention; F - Aligned and Coordinated Interventions (Staff Training); G - Targeted Prevention Intervention; Control Schools reported no changes to their tobacco control programs and policies between Y_1_ and Y_2_.

**Figure 4:**
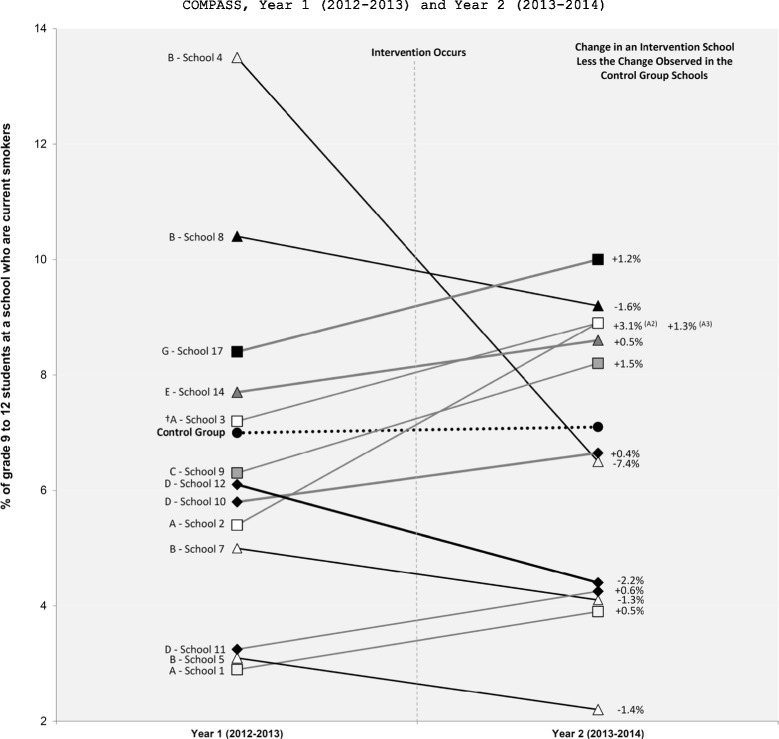
**Difference-in-difference results of the school-level tobacco control programs or policies with significant pre-post differences in the school-level prevalence of current smokers.** † Indicates a program or policy was stopped or removed. A - Media and Social Marketing Interventions; B - Effective and Enforced Tobacco Control Policies; C - Industry Marketing and Promotion Intervention; D - Cessation Interventions; E - Tobacco Denormalization Intervention; G - Targeted Prevention Intervention. Control Schools reported no changes to their tobacco control programs and policies between Y_1_ and Y_2_.

In brief, 14 different schools implemented a new tobacco control program or policy and three schools removed a previously existing tobacco control program or policy. In our follow-up conversations with the 14 schools that implemented a new intervention, only one of them reported that the change was a function of SFO. The changes in the school-level prevalence of susceptible never smokers between Y_1_ and Y_2_ for these 17 intervention schools relative to the control schools are shown in Figure 1. Figure 2 shows the difference-in-difference results for the 6 interventions that resulted in significant Y_1_ to Y_2_ changes in smoking susceptibility prevalence relative to the change observed in the control schools. As shown in Figure 2, only 4 schools implemented interventions that had a positive impact on the prevalence of smoking susceptibility between Y_1_ and Y_2_. The changes in the school-level prevalence of current smoking for these 17 intervention schools relative to the control schools are shown in Figure 3. Figure 4 shows the difference-in-difference results for the 13 interventions that resulted in significant Y_1_ to Y_2_ changes in current smoking prevalence relative to the change observed in the control schools. As shown in Figure 4, only 5 schools implemented interventions that had a positive impact on the prevalence of current smoking between Y_1_ and Y_2_.

#### Media and social marketing interventions

School 1: The school had new tobacco control signage provided from their Public Health Unit (PHU) designed to remind students that they cannot smoke on school property or the property immediately surrounding a school (i.e., the sidewalk at the front of the school off school property). Relative to the control schools, there was not a significant change in the prevalence of smoking susceptibility within this school between Y_1_ and Y_2_, however, there was a significant increase in the prevalence of current smoking within this school between Y_1_ and Y_2_. The within school prevalence of current smokers increased by 30.0% (Figure 3) and the change in the school-level prevalence of current smokers was 0.5% higher than the change observed in the control schools (Figure 4).

School 2: The PHU was in the school for a week to provide an anti-smoking campaign based around a simple collage of pictures showing the negative health effects of smoking. Relative to the control schools, there was not a significant change in the prevalence of smoking susceptibility within this school between Y_1_ and Y_2_, however, there was a significant increase in the prevalence of current smoking within this school between Y_1_ and Y_2_. The within school prevalence of current smokers increased by 64.8% (Figure 3) and the change in the school-level prevalence of current smokers was 3.1% higher than the change observed in the control schools (Figure 4).

School 3: The school stopped allowing the PHU to provide a program that educates youth about the harms of smokeless tobacco use. Relative to the control schools, there was not a significant change in the prevalence of smoking susceptibility within this school between Y_1_ and Y_2_, however, there was a significant increase in the prevalence of current smoking within this school between Y_1_ and Y_2_. The within school prevalence of current smokers increased by 23.6% (Figure 3) and the change in the school-level prevalence of current smokers was 1.3% higher than the change observed in the control schools (Figure 4).

#### Effective and enforced tobacco control policies

School 4: The school implemented a new policy of progressive punishment for students caught smoking on school property (first offense is a discussion with administration, second offense is a fine, third offense is suspension). Relative to the control schools, there was not a significant change in the prevalence of smoking susceptibility within this school between Y_1_ and Y_2_, however, there was a significant decrease in the prevalence of current smoking within this school between Y_1_ and Y_2_. The within school prevalence of current smokers decreased by 51.9% (Figure 3) and the change in the school-level prevalence of current smokers was 7.4% lower than the change observed in the control schools (Figure 4). Interestingly, this school had the largest decline in current smoking rates between Y_1_ and Y_2_ and it was the only school that listed tobacco control as their top priority after receiving the results from the Y_1_ School Health Profile.

School 5: The school implemented a new diversion program to complement their existing policy of progressive punishment for students caught smoking on school property (first offense a student is charged, second offense is a suspension). In the diversion program, a first offense charge can be waived and a second offense suspension can be decreased if the student works with a community liaison officer to complete community service hours. Relative to the control schools, there was not a significant change in the prevalence of smoking susceptibility within this school between Y_1_ and Y_2_, however, there was a significant decrease in the prevalence of current smoking within this school between Y_1_ and Y_2_. The within school prevalence of current smokers decreased by 31.3% (Figure 3) and the change in the school-level prevalence of current smokers was 1.4% lower than the change observed in the control schools (Figure 4).

School 6: The school improved compliance with the ban on smoking on school property and the administration now consistently enforces progressive punishment for students caught smoking on school property (first offense a student is charged, second offense is suspension). Relative to the control schools, there was a significant increase in the prevalence of smoking susceptibility within this school between Y_1_ and Y_2_. The within school prevalence of susceptible never smokers increased by 27.8% (Figure 1) and the change in the school-level prevalence of susceptible never smokers was 6.1% higher than the change observed in the control schools (Figure 2). Relative to the control schools, there was not a significant change in the prevalence of current smoking within this school between Y_1_ and Y_2_

School 7: A new school policy was implemented where a Tobacco Enforcement Officer (TEO) was now involved if students were caught smoking on school property (the specific punishment involved was not articulated in the policy as it seems to be at the discretion of the TEO). Relative to the control schools, there was a significant increase in the prevalence of smoking susceptibility within this school between Y_1_ and Y_2_. The within school prevalence of susceptible never smokers increased by 27.1% (Figure 1) and the change in the school-level prevalence of susceptible never smokers was 5.0% higher than the change observed in the control schools (Figure 2). Relative to the control schools, there was also a significant decrease in the prevalence of current smoking within this school between Y_1_ and Y_2_. The within school prevalence of current smokers decreased by 18.0% (Figure 3) and the change in the school-level prevalence of current smokers was 1.3% lower than the change observed in the control schools (Figure 4).

School 8: A new policy was in place where the PHU was now responsible for sending letters home to parents of students caught smoking on school property. As part of the policy, if a total of 3 letters are sent home (i.e., caught smoking on school property 3 times) the student was also fined $300-400. Relative to the control schools, there was a significant increase in the prevalence of smoking susceptibility within this school between Y_1_ and Y_2_. The within school prevalence of susceptible never smokers increased by 25.7% (Figure 1) and the change in the school-level prevalence of susceptible never smokers was 6.7% higher than the change observed in the control schools (Figure 2). Relative to the control schools, there was also a significant decrease in the prevalence of current smoking within this school between Y_1_ and Y_2_. The within school prevalence of current smokers decreased by 11.5% (Figure 3) and the change in the school-level prevalence of current smokers was 1.6% lower than the change observed in the control schools (Figure 4).

#### Industry marketing and promotion intervention

School 9: The school implemented a new policy that bans students from carrying or wearing clothing and apparel with a tobacco company/product name or logo. Relative to the control schools, there was not a significant change in the prevalence of smoking susceptibility within this school between Y_1_ and Y_2_, however, there was a significant increase in the prevalence of current smoking within this school between Y_1_ and Y_2_. The within school prevalence of current smokers increased by 30.2% (Figure 3) and the change in the school-level prevalence of current smokers was 1.5% higher than the change observed in the control schools (Figure 4).

#### Cessation interventions

School 10: The school reported that they started to provide students with smoking cessation services, although the school could not clearly identify what the specific services were. Relative to the control schools, there was not a significant change in the prevalence of smoking susceptibility within this school between Y_1_ and Y_2_, however, there was a significant increase in the prevalence of current smoking within this school between Y_1_ and Y_2_. The within school prevalence of current smokers increased by 13.8% (Figure 3) and the change in the school-level prevalence of current smokers was 0.4% higher than the change observed in the control schools (Figure 4).

School 11: A tobacco control program managed by a local university and provincial stakeholder organization responsible for school-based tobacco control programming was started in the school. This well-funded 2-year program, as part of Smoke-Free Ontario Strategy (SFO), is based around a youth engagement model to get students with the school to promote smoke-free lifestyles and spaces within their school and among their student peers. Relative to the control schools, there was not a significant change in the prevalence of smoking susceptibility within this school between Y_1_ and Y_2_, however, there was a significant increase in the prevalence of current smoking within this school between Y_1_ and Y_2_. The within school prevalence of current smokers increased by 31.3% (Figure 3) and the change in the school-level prevalence of current smokers was 0.6% higher than the change observed in the control schools (Figure 4).

School 12: Two teachers in this school championed their own tobacco cessation program (program name or details about what the program involved were not provided) to compliment presentations made within the school by a team of student nurses from a local college who developed and presented a smoking prevention program within the school (program name or details about what the program involved were not provided). Relative to the control schools, there was a significant decrease in the prevalence of smoking susceptibility within this school between Y_1_ and Y_2_. The within school prevalence of susceptible never smokers decreased by 13.7% (Figure 1) and the change in the school-level prevalence of susceptible never smokers was 5.5% lower than the change observed in the control schools (Figure 2). Relative to the control schools, there was also a significant decrease in the prevalence of current smoking within this school between Y_1_ and Y_2_. The within school prevalence of current smokers decreased by 29.5% (Figure 3) and the change in the school-level prevalence of current smokers was 2.2% lower than the change observed in the control schools (Figure 4).

School 13: This is the first year where the PHU did not come to the school to lead any form of tobacco cessation programming. Relative to the control schools, there was not a significant change in the prevalence of smoking susceptibility or current smoking within this school between Y_1_ and Y_2_.

#### Tobacco denormalization intervention

School 14: Students were allowed to smoke on school property in a small area in the back of the school, but the location of the smoking area was now moved to the front of the school off school property. Relative to the control schools, there was not a significant change in the prevalence of smoking susceptibility within this school between Y_1_ and Y_2_, however, there was a significant increase in the prevalence of current smoking within this school between Y_1_ and Y_2_. The within school prevalence of current smokers increased by 11.7% (Figure 3) and the change in the school-level prevalence of current smokers was 0.5% higher than the change observed in the control schools (Figure 4).

#### Aligned and coordinated interventions (Staff Training)

School 15: Teachers were now actively encouraged to receive professional development training related to tobacco prevention and to attend conferences, workshops or presentations related to tobacco control. The school did not reported how many teachers actually participated in this additional training. Relative to the control schools, there was a significant increase in the prevalence of smoking susceptibility within this school between Y_1_ and Y_2_. The within school prevalence of susceptible never smokers increased by 26.8% (Figure 1) and the change in the school-level prevalence of susceptible never smokers was 4.2% higher than the change observed in the control schools (Figure 2). Relative to the control schools, there was not a significant change in the prevalence of current smoking within this school between Y_1_ and Y_2_

School 16: Teachers were no longer allowed to receive professional development training related to tobacco prevention using school resources and were no longer allowed to attend conferences, workshops or presentations related to tobacco control using school resources. Relative to the control schools, there was a significant decrease in the prevalence of smoking susceptibility within this school between Y_1_ and Y_2_. The within school prevalence of susceptible never smokers decreased by 11.9% (Figure 1) and the change in the school-level prevalence of susceptible never smokers was 5.7% lower than the change observed in the control schools (Figure 2). Relative to the control schools, there was not a significant change in the prevalence of current smoking within this school between Y_1_ and Y_2_

#### Targeted prevention intervention

School 17: The school created a ‘Traditional Tobacco’ display to provide knowledge and awareness of the sacred use of tobacco among Aboriginals and how there are health risks associated with non-sacred (traditional) use of tobacco. Relative to the control schools, there was not a significant change in the prevalence of smoking susceptibility within this school between Y_1_ and Y_2_, however, there was a significant increase in the prevalence of current smoking within this school between Y_1_ and Y_2_. The within school prevalence of current smokers increased by 19.0% (Figure 3) and the change in the school-level prevalence of current smokers was 1.2% higher than the change observed in the control schools (Figure 4).

## Discussion

Consistent with national estimates [2,3], we identified that a substantial number of youth in this study are either at risk for becoming future smokers or currently smoking. When one considers the national decline in tobacco control as a priority in Canada [24], and that we also identified the paucity of schools participating in COMPASS that identified tobacco prevention to be a prevention priority at their school, this is cause for concern. In fact, even after all participating schools were provided with their school specific results in Y_1_ (results that highlighted there are no schools in the study where tobacco use is not an issue), only one school identified tobacco prevention as a prevention priority in their school in Y_2_, and this only occurred after the school was made aware that at baseline, 13.5% of their students were current smokers (substantially higher than the national average). Interestingly, this school (School 4) reported that once they realized that tobacco use was a real problem among students within the school, they decided to implement a new school specific policy to prevent student smoking at school with the help of the COMPASS knowledge broker. As a result, the prevalence of current smokers at that school was cut in half between Y_1_ and Y_2_. This evidence is consistent with research suggesting that tailoring evidence to the needs of a school can be an effective approach to fostering prevention programming action [25].

Overall, we identified 17 different school-based interventions that took place in 17 different schools between Y_1_ and Y_2_. These 17 interventions were aligned with seven of the different SFO intervention area classifications [12]. Based on the evidence generated here, it appears that the interventions specific to ‘effective and enforced tobacco control’ were not only the most common (5 interventions), but also tended to have the desired impact. Consistent with evidence that strongly enforced school policies designed to prevent students from smoking on school property can be effective at reducing tobacco use among youth [9,26-29], we found that four out of the five schools that implemented new policies to increase the punishment associated with students caught smoking on school property had a significant reduction in current smoking among students between Y_1_ and Y_2_. Three of these five interventions also resulted in what was considered a desirable change in the school-level prevalence of smoking susceptibility between Y_1_ and Y_2_. This is consistent with the tenants of the TTI [4], suggesting that social contexts that would reduce both opportunities to smoke and the normative appeal of smoking should reduce the likelihood of non-smokers transitioning into smoking or increase the likelihood that current smokers quit. Additional research is required to further examine these promising interventions using the longitudinal student-level data.

We also identified three interventions involving teachers that also appear to show some promise. For instance, the teacher-initiated cessation program in School 12 was the only other intervention that had the desired impact on reducing the school-level prevalence of current smoking. Given that we currently have limited detail on the specific intervention components or approaches that were used within this school beyond the information provided in the SPP, there would be substantial value in additional follow-up research with the teachers leading this intervention to determine what specific cessation activities took place and to then further evaluate the intervention impact with the longitudinal student-level data. Considering that this school also had a significant decline in the prevalence of susceptible never smokers between Y_1_ and Y_2_, exploring if this intervention had an impact on reducing smoking uptake with the longitudinal student-level data seems warranted. Similarly, two schools reported interventions associated with ‘alignment and coordination (via staff training in tobacco control)’; School 15 encouraged teachers to participate in additional tobacco control and School 16 that prohibited teachers from participating in additional tobacco control training. Not surprisingly, the desired impact on susceptibility was evident when the intervention promoted teacher training and the undesired impact on susceptibility was evident in the intervention that discouraged teacher training. Consistent with evidence highlighting the importance of training on tobacco control specific system outcomes [30], this appears to be indicative that such training may be an important and currently underutilized opportunity in school-based tobacco control programming.

A cause for concern is that many of the interventions identified and examined here actually appear to have had an undesirable impact on the school-level prevalence of smoking susceptibility and/or current smoking. For instance, although the Ontario Public Health Standards (OPHS) contain a requirement that boards of health shall work with schools to develop and implement tobacco control programming within schools [11], two of the interventions that involved having public health staff in schools (School 1, School 2) actually appear to have had a negative outcome where school-level rates of current smoking increased significantly. Two of the interventions focused on cessation programming (School 10, School 11) also appear to have had a negative outcome where school-level rates of current smoking increased significantly. While this may not be surprizing in School 10 where the intervention was not evidence-based or well-coordinated, the outcome observed in School 11 is cause for concern as this intervention is currently provincially supported. Other interventions with outcomes for current smoking that raise concern include the intervention in School 9 (preventing youth from wearing tobacco specific merchandise) and School 17 (teaching students about the traditional and non-traditional uses of tobacco). Additional research is required to better understand what was involved in the implementation of these interventions, and to understand why they may have had a negative outcome despite being aligned with prevention recommendations currently supported in Ontario [12].

According to Government of Ontario [11], a key to the success of SFO within school settings requires that those involved need to: work with local health departments to coordinate school-based activities; work with schools to implement evidence-based tobacco prevention programming; and, work with schools to develop stronger tobacco control policies and enforcement strategies. These data illustrate that thus far, COMPASS has had some success in accomplishing all three of these goals [18]. However, these data also indicate that there are additional partnerships that should be made between schools and PHUs, local institutions, and provincial stakeholders to further support the development, implementation and evaluation of tobacco prevention programs. Only 7 school contacts explicitly indicated that they had worked with other organizations to develop tobacco programs either currently or in the past, but in two of these schools partnerships ended. These data also show that there are schools (e.g., School 12, School 17) that are willing to improve their tobacco programming but may not have adequate knowledge of evidence-based practices or the appropriate tools to effectively plan and implement interventions. It is clear that partnerships between schools and local tobacco control experts are necessary to promote the exchange of evidence-based interventions, especially considering the lack of available school-based prevention resources available within Ontario at the present time. Furthermore, a more inclusive partnership between COMPASS, the Government of Ontario, and SFO stakeholders (e.g., the Ontario Tobacco Research Unit (OTRU)) would help to generate the evidence necessary to maximize the potential success of future provincial tobacco control prevention initiatives.

The study design used in COMPASS provides both robust internal control (at the student- and school-level as a function of the longitudinal design) when examining change over time, and robust external validity (as a function of the quasi-experimental design when evaluating real-world interventions) [31]. For instance, given that lack of available evidence about what anti-tobacco programs are effective within schools [9,10], it may be more appropriate and feasible to recommend the promising interventions identified here to schools with similar social or cultural contexts [4]. Similarly, it is important to recommend to schools to stop or delay the interventions that appear to be having a deleterious impact. Although the strength of the evidence pertaining to the promising programs identified here is not perfect, these programs represent emerging real-world practice-based evidence that now warrants additional investigation [15]. Given that this study has identified seven promising programs via this evaluation (two that had the desired impact on both smoking susceptibility and current smoking, two that only had the desired impact on smoking susceptibility, and three that only had the desired impact on current smoking), additional research should now examine these programs in more detail using the longitudinal student-level data, controlling for relevant student- and school-level correlates.

A limitation of the present study was that no data were available from the SPP pertaining to process or implementation issues for each of the 17 interventions identified (e.g., how consistently were policies enforced, program fidelity, etc.). Although such intervention process detail is well beyond the scope of this manuscript, the evidence presented here clearly helps to identify promising tobacco control interventions that occurred within COMPASS between Y_1_ and Y_2_ that require additional investigation. Moving forward, we can now compile and collect additional intervention data from the administrators in the schools that implemented interventions with an apparent positive impact on student smoking (e.g., Schools 7 and 8 as shown in Figures 2 and 4) to perform more substantive evaluation research (e.g., how particular policies were implemented and/or enforced, what staff and financial resources were required, external partnerships, duration of the intervention, etc.). It is also possible that a decline in the school-level prevalence of smoking susceptibility may be a positive program outcome (i.e., the intervention caused susceptible never smokers to no longer be susceptible). Although it cannot be determined with the data examined here, the interventions in School 12 and 16 warrant further investigation with the linked longitudinal student-level data. Similarly, since these data also generalize outcomes at the school-level, they do not account for changes in smoking behaviours between incoming and outgoing student cohorts which may impact the results. However, as additional data points for the longitudinal student-level data are available in COMPASS (Year 3, Year 4), longitudinal hierarchical can be used to examine how each of the promising interventions identified here are related to changes in the individual student-level smoking outcomes over time, controlling for individual and school-level correlates. These longitudinal examinations can also examine the potential differential impact of such interventions on sub-populations of at-risk youth within the schools (e.g., among the off-reserve Aboriginal youth, youth with co-occurring substance use) [3,32].

An additional limitation of this study is that COMPASS relies on self-reports of smoking behaviour, so the findings may reflect some under-reporting bias which is common in youth smoking research. However, COMPASS data are based on previously validated self-reported measures of youth smoking [20,21] and honest reporting was encouraged by ensuring confidentiality during data collection. Participation bias is also mitigated by not informing eligible students of the specific data collection data ahead of time. Given that COMPASS data are longitudinal, potential bias in the self-reported data is also partially mitigated as any over or under-reporting bias should be consistent over time [31]. It is also important to note that this study did not examine changes to the prevalence of smoking stages (experimentation, former smokers) [1], or the use of other tobacco products (e.g., hookah, cigarillos, *e*-cigarettes, etc.) between Y_1_ and Y_2_. Including additional outcome categories or tobacco products was beyond the scope of this manuscript. Future research should duplicate this work examining those other tobacco-related outcomes, as well as duplicating these methods for examining the impact of school-based prevention programming in other behavioural domains measured in COMPASS (e.g., physical activity, diet, obesity, alcohol use, and bullying).

## Conclusion

Natural experiments, such as the 17 interventions examined here within COMPASS, may represent the best currently available real-world public health evidence pertaining to identifying new promising tobacco control interventions among youth [16]. Clearly we need to develop more effective methods of identifying effective interventions in youth tobacco control as the available evidence-base of how to effectively intervene is limited [9,10]. Progress in preventing smoking onset or promoting cessation among current smoking youth will require efforts from many different stakeholders in many different contexts, and the use of innovative methods for identifying which interventions work, for whom, and in which context [14]. The evidence presented here is indicative that some school-based tobacco control interventions appear promising for potentially preventing smoking uptake and that data collection systems such as COMPASS can provide the infrastructure to support this valuable practice-based evidence.

## References

[1] Mayhew KP, Flay BR, Mott JA (2000). Stages in the development of adolescent smoking. Drug Alcohol Depend.

[2] Kaai S, Brown KS, Leatherdale ST, Manske KS, Murnaghan D (2014). We do not smoke but some of us are more susceptible than others: a multilevel analysis of a sample of Canadian youth in grades 9 to 12. Add Behav.

[3] Leatherdale ST, Rynard V (2013). A cross-sectional examination of modifiable risk factors for chronic disease among a nationally representative sample of youth: are Canadian students graduating high school with a failing grade for health?. BMC Public Health.

[4] Flay BR (1999). Understanding environmental, situational and interpersonal risk and protective factors for youth tobacco use: the Theory of Triadic Influence. Nicotine Tob Res.

[5] U.S. Department of Health and Human Services (2012). Preventing tobacco Use among youth and young adults: a report of the surgeon general.

[6] Leatherdale ST, Brown KS, Cameron R, McDonald PW (2005). Social modelling in the school environment, student characteristics, and smoking susceptibility: a multi-level analysis. J Adolesc Health.

[7] Kaai S, Leatherdale ST, Manske KS, Brown KS (2013). Using student and school factors to differentiate adolescent current smokers from experimental smokers in Canada: a multilevel analysis. Prev Med.

[8] Leatherdale ST, McDonald PW, Cameron R, Brown KS (2005). A multi-level analysis examining the relationship between social influences for smoking and smoking onset. Am J Health Behav.

[9] Galanti MR, Coppo A, Jonsson E, Bremberg S, Faggiano F (2014). Anti-tobacco policy in schools: upcoming preventive strategy or prevention myth? A review of 31 studies. Tob Control.

[10] Wiehe SE, Garrison MM, Christakis DA, Ebel BE, Rivara FP (2005). A systematic review of school-based smoking prevention trials with long-term follow-up. J Adolesc Health.

[11] Ministry of Health Promotion: Comprehensive Tobacco Control: Guidance Document. Queen’s Printer for Ontario, 2010. (ISBN: 978-1-4435-2910-5). Available at: http://www.mhp.gov.on.ca/en/healthy-communities/public-health/guidance-docs/ComprehensiveTobaccoControl.PDF Accessed on August 9, 2014.

[12] Smoke-Free Ontario - Scientific Advisory Committee (2010). Evidence to guide action: comprehensive tobacco control in Ontario.

[13] Ontario Tobacco Research Unit: Smoke-Free Ontario Strategy Monitoring Report. Toronto: Ontario Tobacco Research Unit, Special Report, January 2014. Available at: http://otru.org/wp-content/uploads/2014/02/OTRU-SMR-2013.pdf Accessed on August 9, 2014.

[14] Leatherdale ST (2012). Evaluating school-based tobacco control programs and policies: an opportunity gained and many opportunities lost. Can J Prog Eval.

[15] Green LW (2006). Public health asks of systems science: to advance our evidence-based practice, can you help us get more practice-based evidence?. Am J Public Health.

[16] Petticrew M, Cummins S, Ferrell C, Findlay A, Higgins C, Hoy C (2005). Natural experiments: an underused tool for public health?. Public Health.

[17] Cameron R, Manske S, Brown KS, Jolin MA, Murnaghan D, Lovato C (2007). integrating public health policy, practice, evaluation, surveillance, and research: the school health action planning and evaluation system. Am J Public Health.

[18] Leatherdale ST, Brown KS, Carson V, Childs RA, Dubin JA, Elliott SJ (2014). The COMPASS study: a longitudinal hierarchical research platform for evaluating natural experiments related to changes in school-level programs, policies and built environment resources. BMC Public Health.

[19] Marcus SE, Leischow SJ, Mabry PL, Clark PI (2010). Systems science: a revolution in public health policy research. Am J Public Health.

[20] Wong S, Shields M, Leatherdale ST, Malaison E, Hammond D (2012). Assessment of the validity of self-reported smoking status among Canadians. Health Rep.

[21] Pierce JP, Choi WS, Gilpin EA, Farkas AJ, Merritt RK (1996). Validation of susceptibility as a predictor of which adolescents take up smoking in the United States. Health Psych.

[22] Pan Canadian Joint Consortium for School Health (2014). Healthy School Planner.

[23] Shadish WR, Cook TD, Campbell DT (2002). Experimental and quasi-experimental designs for generalized causal inference.

[24] Picard A. Is tobacco control no longer a federal priority? The Globe and Mail. May 25, 2011. Available at: http://www.theglobeandmail.com/life/health-and-fitness/is-tobacco-control-no-longer-a-federal-priority/article624882/ Accessed on August 06, 2014.

[25] Cameron R, Brown KS, Best JA, Pelkman CL, Madill CL, Manske SR (1999). Effectiveness of a social influences smoking prevention program as a function of provider type, training method, and school risk. Am J Public Health.

[26] Lovato CY, Pullman AW, Halpin P, Zeisser C, Nykiforuk CI, Best F (2010). The influence of school policies on smoking prevalence among students in grades 5–9, Canada, 2004–2005. Prev Chronic Dis.

[27] Pinilla J, Gonzalez B, Barber P, Santana Y (2002). Smoking in young adolescents: an approach with multilevel discrete choice models. J Epi Comm Health.

[28] Moore L, Roberts C, Tudor-Smith C (2001). School smoking policies and smoking prevalence among adolescents: multilevel analysis of cross-sectional data from Wales. Tob Control.

[29] Wakefield MA, Chaloupka FJ, Kaufman NJ, Orleans CT, Barker DC, Ruel EE (2000). Effect of restrictions on smoking at home, at school and in public places on teenage smoking: cross sectional study. BMJ.

[30] Leatherdale ST, Viehbeck S, Murphy C, Norman C, Schultz A (2007). The tobacco control community of tomorrow: a vision for training. Can J Public Health.

[31] Diggle PJ, Liang K-L, Zeger SL (2002). Analysis of longitudinal data.

[32] Elton-Marshall T, Leatherdale ST, Burkhalter R, Brown KS (2013). Changes in tobacco use, susceptibility to future smoking, and quit attempts among Canadian youth over time: a comparison of off-reserve Aboriginal and non-Aboriginal youth. Int J Env Res Public Health.

